# The influence on survival of delay in the presentation and treatment of symptomatic breast cancer

**DOI:** 10.1038/sj.bjc.6690137

**Published:** 1999-02

**Authors:** M A Richards, P Smith, A J Ramirez, I S Fentiman, R D Rubens

**Affiliations:** ICRF Clinical and Psychosocial Oncology Groups, GKT School of Medicine, St Thomas' Hospital, London, SE1 7EH, UK

**Keywords:** breast cancer, delay, duration of symptoms, survival

## Abstract

The aim of this study was to examine the possible influence on survival of delays prior to presentation and/or treatment among women with breast cancer. Duration of symptoms prior to hospital referral was recorded for 2964 women who presented with any stage of breast cancer to Guy's Hospital between 1975 and 1990. Median follow-up is 12.5 years. The impact of delay (defined as having symptoms for 12 or more weeks) on survival was measured from the date of diagnosis and from the date when the patient first noticed symptoms to control for lead-time bias. Thirty-two per cent (942/2964) of patients had symptoms for 12 or more weeks before their first hospital visit and 32% (302/942) of patients with delays of 12 or more weeks had locally advanced or metastatic disease, compared with only 10% (210/2022) of those with delays of less than 12 weeks (*P*< 0.0001). Survival measured both from the date of diagnosis (*P*< 0.001) and from the onset of the patient's symptoms (*P*= 0.003) was worse among women with longer delays. Ten years after the onset of symptoms, survival was 52% for women with delays less than 12 weeks and 47% for those with longer delays. At 20 years the survival rates were 34% and 24% respectively. Furthermore, patients with delays of 12–26 weeks had significantly worse survival rates than those with delays of less than 12 weeks. Multivariate analyses indicated that the adverse impact of delay in presentation on survival was attributable to an association between longer delays and more advanced stage. However, within individual stages, longer delay had no adverse impact on survival. Analyses based on ‘total delay’ (i.e. the interval between a patient first noticing symptoms and starting treatment) yielded very similar results in terms of survival to those based on delay to first hospital visit (delay in presentation). © 1999 Cancer Research Campaign


*British Journal of Cancer* (1999) **79**(5/6), 858–864
© 1999 Cancer Research Campaign
Article no. bjoc.1998.0137

### The influence on survival of delay in the presentation and treatment of symptomatic breast cancer

**MA Richards, P Smith, AJ Ramirez, IS Fentiman and RD Rubens**

ICRF Clinical and Psychosocial Oncology Groups, GKT School of Medicine, St Thomas' Hospital, London SE1 7EH, UK

**Summary** The aim of this study was to examine the possible influence on survival of delays prior to presentation and/or treatment among women with breast cancer. Duration of symptoms prior to hospital referral was recorded for 2964 women who presented with any stage of breast cancer to Guy's Hospital between 1975 and 1990. Median follow-up is 12.5 years. The impact of delay (defined as having symptoms for 12 or more weeks) on survival was measured from the date of diagnosis and from the date when the patient first noticed symptoms to control for lead-time bias. Thirty-two per cent (942/2964) of patients had symptoms for 12 or more weeks before their first hospital visit and 32% (302/942) of patients with delays of 12 or more weeks had locally advanced or metastatic disease, compared with only 10% (210/2022) of those with delays of less than 12 weeks ($P < 0.0001$). Survival measured both from the date of diagnosis ($P < 0.001$) and from the onset of the patient's symptoms ($P = 0.003$) was worse among women with longer delays. Ten years after the onset of symptoms, survival was 52% for women with delays less than 12 weeks and 47% for those with longer delays. At 20 years the survival rates were 34% and 24% respectively. Furthermore, patients with delays of 12–26 weeks had significantly worse survival rates than those with delays of less than 12 weeks. Multivariate analyses indicated that the adverse impact of delay in presentation on survival was attributable to an association between longer delays and more advanced stage. However, within individual stages, longer delay had no adverse impact on survival. Analyses based on 'total delay' (i.e. the interval between a patient first noticing symptoms and starting treatment) yielded very similar results in terms of survival to those based on delay to first hospital visit (delay in presentation).

**Keywords:** breast cancer; delay; duration of symptoms; survival

More than 50% of all patients who present with breast cancer ultimately die of the disease. The use of systemic adjuvant therapy yields modest improvements in prognosis, which should translate into a considerable number of lives saved, because of the high incidence of breast cancer (Early Breast Cancer Trialists' Collaborative Group, 1992). Other measures are, however, required if the number of deaths is to be decreased substantially. Research into possible methods of prevention and cure will hopefully yield significant benefits in the longer term. For the present, however, earlier detection and treatment offers the best chance of reducing mortality.

Extent of disease (stage) at diagnosis is widely recognized to be the most important prognostic factor in patients with breast cancer. The aim of the National Health Service Breast Screening Programme (NHSBSP) is to detect breast cancer at a presymptomatic stage and thus to improve survival. However, most women with breast cancer present with symptoms (Macarthur and Smith, 1981; Burgess et al, 1998). These patients may seek medical advice soon after the discovery of symptoms or may delay presenting to their general practitioner (GP) ('patient delay'). Delays may also occur between the first GP visit and first hospital visit or between the first hospital visit and treatment. The influence on survival of delays in each of these intervals and of the influence on survival of delays between first symptom and treatment ('total delay') remains contentious. Several studies reported in the past 30 years have indicated that survival is worse among women with longer duration of symptoms (Sheridan et al, 1971; Wilkinson et al, 1979; Elwood and Moorehead, 1980; Feldman et al, 1983; Charlson, 1985; Vernon et al, 1985; Huguley et al, 1988; Machiavelli et al, 1989; Rossi et al, 1990; Neave et al, 1990; Rabinovich et al, 1993; Afzelius et al, 1994; Raabe et al, 1996). Other studies, however, have not shown that survival is affected by duration of symptoms (Dennis et al, 1975; Fisher et al, 1977; Hainsworth et al, 1993) These apparently conflicting results may possibly be explained by differences in sample characteristics (e.g. inclusion of patients with all stages of breast cancer or restriction of the sample to patients with operable disease only), by differences in the delay interval studied (e.g. patient delay, delay to first hospital visit or total delay) or by differences in the cut-offs used to define delay (e.g. 3 months or 6 months).

A major problem with most of the previously reported studies of the influence of delay on survival is that no account has been taken of the potential confounding effect of lead-time bias. According to the null hypothesis, earlier treatment confers no survival advantage over later treatment among patients who present with symptomatic disease. The null hypothesis implies that some patients will be long-term survivors while others will die of their disease, irrespective of the time of first treatment. For those who are predestined to die, the interval between treatment and death will be shorter if treatment is started later in the course of the disease. An apparent adverse influence of delay on survival could thus be attributable to this lead-time effect if survival is measured from the time of diagnosis. Measurement of survival from the time that a patient first notices symptoms, rather than from the time of diagnosis (as is conventionally reported), addresses this issue.

*Received 28 October 1997*
*Revised 2 June 1998*
*Accepted 8 July 1998*

*Correspondence to:* MA Richards, Department of Palliative Medicine, St Thomas' Hospital, London, UK

**Table 1** Proportion of patients experiencing delays in presentation and/or treatment

| | Onset of symptoms to first hospital visit | | First hospital visit to treatment | | Onset of symptoms to treatment | |
|---|---|---|---|---|---|---|
| | *n* | (%) | *n* | (%) | *n* | (%) |
| < 1 week | 284 | (10) | 1496 | (51) | 36 | (1) |
| 1–2 weeks | 407 | (14) | 661 | (23) | 152 | (5) |
| 2–3 weeks | 291 | (10) | 338 | (12) | 282 | (10) |
| 3–4 weeks | 349 | (12) | 176 | (6) | 288 | (10) |
| 4–8 weeks | 572 | (19) | 238 | (8) | 822 | (28) |
| 8–12 weeks | 119 | (4) | 21 | (1) | 372 | (13) |
| 12–26 weeks | 529 | (18) | 3 | (<1) | 491 | (17) |
| 26–52 weeks | 211 | (7) | 1 | (<1) | 204 | (7) |
| > 52 weeks | 202 | (7) | 3 | (<1) | 290 | (10) |
| Total | 2964 | | 2937 | | 2937 | |

In this study, we have assessed the relationship between duration of symptoms and survival among almost 3000 women managed in a single institution between 1975 and 1990. Our primary hypothesis was that, when all patients presenting with breast cancer are considered, those with duration of symptoms of at least 12 weeks would have worse survival rates than those with shorter delays. A number of secondary hypotheses were also defined: first, that patients with longer duration of symptoms would in general present with more advanced disease and that this relationship between delay and stage would account for the poorer survival in patients with longer delays; and, second, that for patients within any individual tumour stage longer delays would have no detrimental effect on survival. To illustrate this, consider two patients, each presenting with a 1.5-cm, node-negative (stage I) cancer, one of whom has had a 2-week delay, the other a 9-month delay. According to this secondary hypothesis the patient with the 9-month delay would have at least as good a prognosis as the patient with the shorter delay. This effect has previously been reported for patients with stage I cancer (Sheridan, 1971; Wilkinson, 1979; Charlson, 1985) and for patients with stage III tumours (Rubens et al, 1977).

### METHODS

### Patients

Computerized records of all patients with breast cancer who were referred directly to the breast unit at Guy's Hospital between 1 January 1975 and 31 December 1990 were reviewed. Patients who had initially been diagnosed elsewhere and had subsequently been referred to the unit for further management were excluded. The study period was selected because uniform criteria for histological assessment and staging had been used throughout. The end date of the study was chosen to give a minimum of 5 years' follow-up for all patients. In addition, virtually all patients presented symptomatically during this period, whereas more recently a significant number of patients have presented via the NHSBSP.

### Data collection

At the time of the first hospital visit all patients were asked to complete a proforma, which included a question on duration of symptoms. Other factors retrieved from the computer database included date of first hospital attendance, date of diagnosis, date of first definitive treatment, age and menopausal status at diagnosis, date of last follow-up and date of death (where applicable). Information on clinical tumour size, histological type and grade (Bloom and Richardson, 1957), pathological axillary node status and stage was also extracted from the database. The staging classification used was as follows:

stage I – operable disease with pathologically negative axillary nodes;
stage II – operable disease with pathologically positive nodes;
stage III – locally advanced, inoperable disease;
stage IV – metastatic disease at presentation.

### Management

Standardized protocols for the assessment and treatment of breast cancer were used within the unit throughout the study period, although these evolved over time. Where appropriate, patients were entered into clinical trials related to the management of the primary tumour (van Dongen et al, 1991) and to the use of systemic therapies (Rubens et al, 1980, 1983, 1989; Nolvadex Adjuvant Trial Organisation, 1988; Richards et al, 1990; Scottish Cancer Trials Breast Group, 1993; Fentiman et al, 1994). In over 90% of all operable cases, histological assessment of axillary lymph node status was undertaken, usually following full axillary dissection.

### Statistical analysis

Two delay intervals were examined for the analyses of the influence of delay on survival. The first was the interval between a patient first noticing symptoms (onset of symptoms) and the first visit to the hospital (defined as 'delay in presentation'). The second was the interval between onset of symptoms and first definitive treatment (defined as 'total delay'). For each of these analyses, arbitrary cut-off points of 12 weeks and 26 weeks were used to define different delay groups for comparability with other reported studies. Patients for whom the recorded duration of symptoms at first hospital visit was either '3 months' or '6 months' were included in the 12–26 week group for the analysis of delay in presentation. Possible relationships between delay and other factors were assessed using the chi-squared test.

The log rank test was used to assess the influence on survival of delay in presentation and total delay in univariate analyses.

© Cancer Research Campaign 1999

**Table 2** Patient and tumour characteristics according to duration of symptoms prior to first hospital visit

| | < 12 weeks | | ≥ 12 weeks | |
|---|---|---|---|---|
| | *n* | (%) | *n* | % |
| *Age* | | | | |
| <35 | 74 | (4%) | 31 | (3%) |
| 35–49 | 555 | (27%) | 212 | (23%) |
| 50–64 | 841 | (42%) | 376 | (40%) |
| 65–74 | 420 | (21%) | 231 | (25%) |
| 75+ | 132 | (7%) | 92 | (10%) |
| *Tumour size*[a] | | | | |
| ≤ 2 cm | 664 | (33%) | 196 | (22%) |
| > 2 cm and ≤ 5 cm | 1126 | (56%) | 466 | (51%) |
| > 5 cms | 203 | (10%) | 245 | (27%) |
| Unknown | 29 | | 35 | |
| *Stage*[b] | | | | |
| Operable $N_0$ | 880 | (44%) | 281 | (30%) |
| Operable $N^+$ | 783 | (39%) | 290 | (31%) |
| Operable N? | 149 | (7%) | 69 | (7%) |
| Locally advanced | 161 | (8%) | 201 | (21%) |
| Metastatic | 49 | (2%) | 101 | (11%) |
| *Histology* | | | | |
| Ductal I | 130 | (6%) | 66 | (7%) |
| Ductal II | 748 | (37%) | 377 | (40%) |
| Ductal III | 615 | (30%) | 230 | (24%) |
| Lobular | 218 | (11%) | 105 | (11%) |
| Other | 311 | (15%) | 164 | (17%) |
| *Year of diagnosis* | | | | |
| 1975–78 | 460 | (23%) | 212 | (23%) |
| 1979–82 | 536 | (27%) | 242 | (26%) |
| 1983–86 | 501 | (25%) | 277 | (29%) |
| 1987–90 | 525 | (26%) | 211 | (22%) |
| Total | 2022 | (68%) | 942 | (32%) |

[a]Tumour size was measured clinically at the time of first hospital visit. [b]Nodal status was categorized as follows: $N_0$, No pathological axillary node involvement. $N^+$, Pathological axillary node involvement. N?, Axillary nodes not examined pathologically.

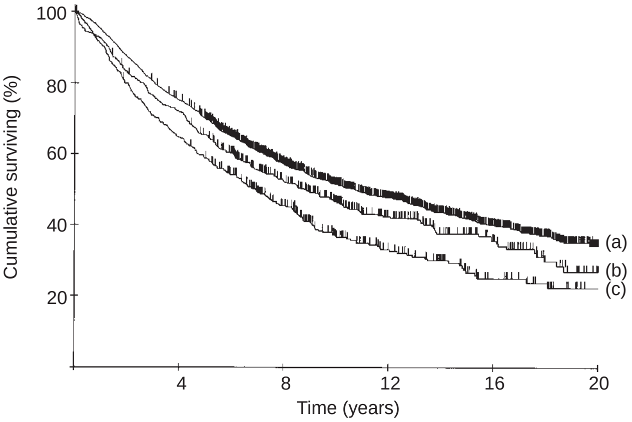

**Figure 1** Survival measured from date of diagnosis, all patients. (a) Delay < 12 weeks ($n$ = 2022). (b) Delay 12–26 weeks ($n$ = 691). (c) Delay > 26 weeks ($n$ = 413). $P$ < 0.0001

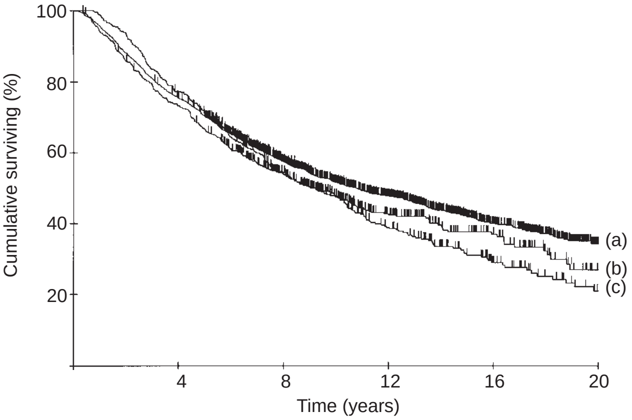

**Figure 2** Survival measured from onset of symptoms, all patients. (a) Delay < 12 weeks ($n$ = 2022). (b) Delay 12–26 weeks ($n$ = 691). (c) Delay > 26 weeks ($n$ = 413). $P$ = 0.003

Survival was measured in two ways: first, from the date of histological diagnosis and, second, from the calculated date of onset of symptoms. The survival analyses relate to all-cause mortality unless otherwise specified.

Multivariate analyses were undertaken using the stepwise Cox regression model with survival from diagnosis and from onset of symptoms as the outcome measures. Age, duration of symptoms prior to first hospital visit, grade and menopausal status (1–5 years post-menopausal vs other) were first included in the model. Similar results were obtained when duration of symptoms was considered as a continuous variable and when the cut-off of 12 weeks was applied. A second set of analyses was undertaken, including clinical tumour size and stage in the model.

### RESULTS

A total of 3099 women with primary breast cancer were referred directly to the unit over the 16-year period of the study. Prospectively recorded information on duration of symptoms prior to first hospital visit was available for 2964 (96%) of these women. No difference in survival was observed between those with or without data on duration of symptoms ($P$ = 0.09). Median survival was 9.5 years. Dates of first hospital visit and date of first treatment were available in 2937 cases (99%). The proportions of patients experiencing different delays prior to presentation, between presentation and treatment and total delays are shown in Table 1.

### Relationship between delay in presentation and tumour characteristics

The relationships between duration of symptoms prior to first hospital visit and other demographic or tumour-related factors are shown in Table 2. Delay was highly significantly related to age, tumour size and stage. Patients over 65 years of age tended to have longer duration of symptoms ($P$ < 0.0001). Among patients with tumours measuring 2 cm or less in diameter, only 196/860 (23%) delayed for 12 weeks or more compared with 466/1592 (29%) of patients with tumours between 2 and 5 cm and 245/448 (55%) of patients with tumours more than 5 cm in diameter ($P$ < 0.0001). Only 640/2452 (26%) of patients with operable disease had symptoms for 12 weeks or more, compared with 302/512 (59%) of patients with locally advanced or metastatic disease ($P$ < 0.0001).

© Cancer Research Campaign 1999

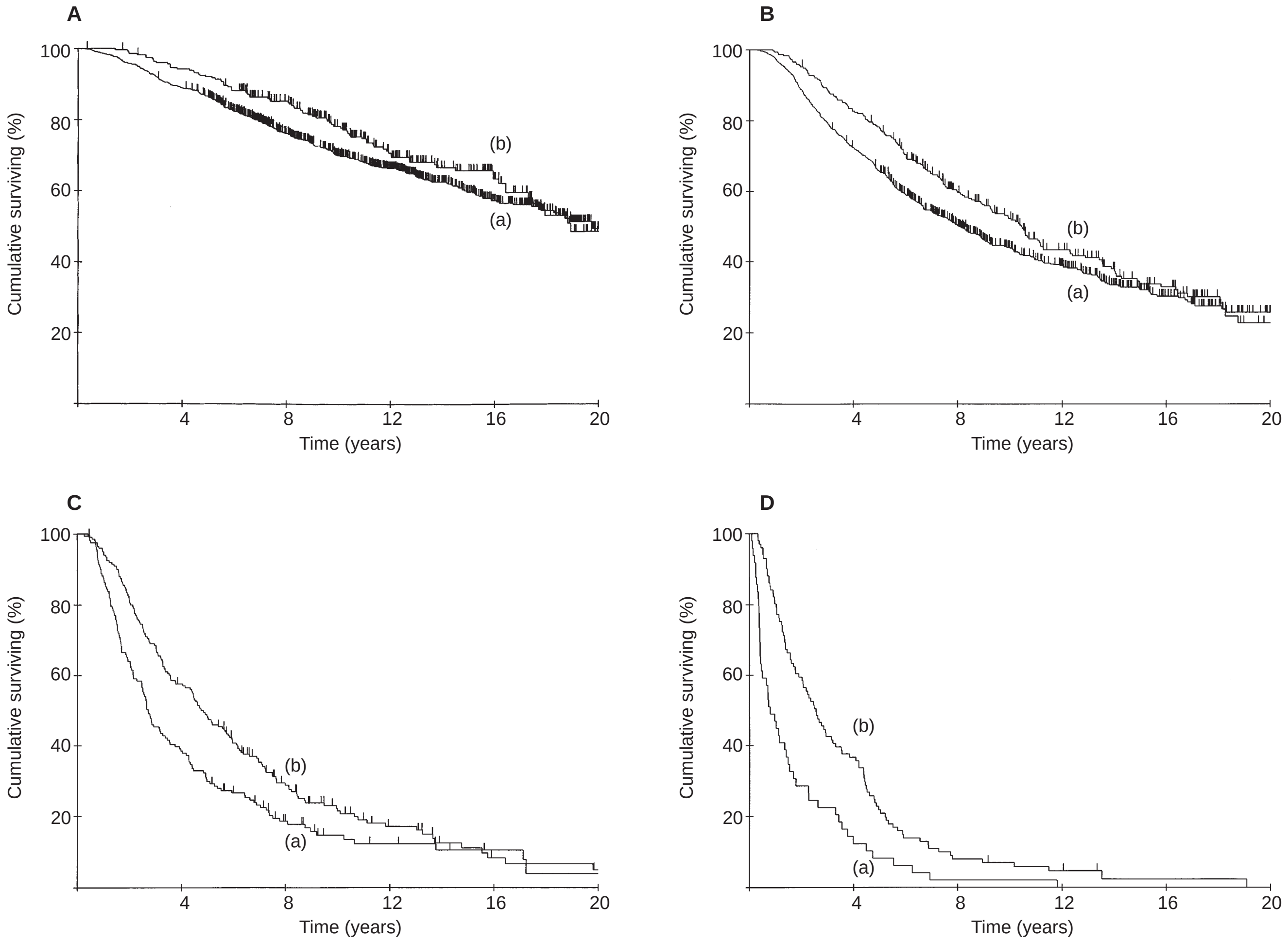

**Figure 3** Survival measured from onset of symptoms (**A**) Stage I. (a) Delay < 12 weeks (*n* = 880). (b) Delay > 12 weeks (*n* = 281). *P* = 0.1. (**B**) Stage II. (a) Delay < 12 weeks (*n* = 880). (b) Delay ≥ 12 weeks (*n* = 281). *P* = 0.03. (**C**) Stage III. (a) Delay < 12 weeks (*n* = 161). (b) Delay ≥ 12 weeks (*n* = 201). *P* < 0.02. (**D**) Stage IV. (a) Delay < 12 weeks (*n* = 49). (b) Delay ≥ 12 weeks (*n* = 101). *P* < 0.001.

Patients with ductal grade III tumours had significantly shorter periods of delay than others ($P < 0.001$). Duration of symptoms did not change significantly over the course of the study period.

Among patients with duration of symptoms prior to first hospital visit of less than 12 weeks, an earlier cut-off (4 weeks) was also examined. No significant differences in the distribution of demographic or tumour characteristics were observed between patients with a delay of 4 weeks or less and those with a delay longer than 4 weeks but less than 12 weeks (data not shown).

### Survival measured from date of diagnosis

Survival measured from the date of diagnosis according to delay in presentation is shown in Figure 1. Longer delay in presentation was significantly associated with worse survival ($P < 0.0001$). At 10 years following diagnosis the *all-cause* survival rates for different delay groups were as follows: 51% (delay < 12 weeks); 44% (delay 12–26 weeks) and 40% (delay > 26 weeks). At 20 years, the survival rates were 33%, 26% and 24% respectively. Thus, a 7% difference in survival between those with short (< 12 weeks) and intermediate (12–26 weeks) delays was observed both at 10 years and at 20 years. When *breast cancer* mortality only was considered, the comparable survival rates at 10 years were 58%, 51% and 47% respectively, and those at 20 years were 48%, 40% and 32% respectively.

### Survival measured from onset of symptoms

Survival measured from the calculated date of onset of symptoms according to delay in presentation is shown in Figure 2. The impact of delay in presentation on survival remains highly significant ($P = 0.003$), but the curves only start to diverge markedly after about 4 years. At 10 years from onset of symptoms a 5% difference in survival was observed between patients with delays of less or more than 12 weeks (52% vs 47%). At 20 years, the difference in survival was 10% (34% vs 24%). Again, when deaths from causes other than breast cancer were excluded, similar survival differences according to delay were observed. At 10 years, the survival rates were 57% and 53% respectively, and at 20 years they were 48% and 33%.

### Subanalyses by stage and grade

Because of the influence of delay on stage, additional analyses were undertaken to assess the influence of delay in presentation on survival within each stage. When survival was measured from the

*© Cancer Research Campaign 1999*

**Table 3** Multivariate analyses of factors influencing survival, excluding factors related to extent of disease (tumour size and stage)

| Variable | Hazard ratio | 95% CI | *P*-value |
|---|---|---|---|
| *Survival measured from histological diagnosis* | | | |
| Grade | 1.65 | 1.51–1.81 | < 0.0001 |
| Duration of symptoms | 1.31 | 1.18–1.45 | < 0.0001 |
| Menopausal status | 1.36 | 1.16–1.59 | < 0.0001 |
| Age | 1.03 | 1.02–1.03 | < 0.0001 |
| *Survival measured from onset of symptoms* | | | |
| Grade | 1.66 | 1.52–1.82 | < 0.0001 |
| Duration of symptoms | 1.15 | 1.04–1.27 | < 0.0001 |
| Menopausal status | 1.37 | 1.17–1.59 | < 0.0001 |
| Age | 1.03 | 1.02–1.03 | < 0.0001 |

Factors directly related to extent of disease (e.g. tumour size and stage) were excluded from this analysis. The hazard ratios indicate that in this analysis longer duration of symptoms is associated with worse survival rates and that this effect is independent of tumour grade, menopausal status and age.

**Table 4** Multivariate analyses of factors influencing survival, including factors related to extent of disease (tumour size and stage)

| Variable | Hazard ratio | 95% CI | *P*-value |
|---|---|---|---|
| *Survival measured from histological diagnosis* | | | |
| Stage | 1.62 | 1.55–1.70 | < 0.0001 |
| Grade | 1.54 | 1.40–1.69 | < 0.0001 |
| Menopausal status | 1.36 | 1.16–1.59 | < 0.0001 |
| Tumour size | 1.09 | 1.07–1.12 | < 0.0001 |
| Age | 1.02 | 1.01–1.02 | < 0.0001 |
| Duration of symptoms | 0.87 | 0.77–0.97 | 0.01 |
| *Survival measured from onset of symptoms* | | | |
| Stage | 1.60 | 1.52–1.68 | < 0.0001 |
| Grade | 1.55 | 1.42–1.70 | < 0.0001 |
| Menopausal status | 1.40 | 1.20–1.64 | < 0.0001 |
| Tumour size | 1.07 | 1.05–1.10 | < 0.0001 |
| Age | 1.02 | 1.01–1.02 | < 0.0001 |
| Duration of symptoms | 0.72 | 0.64–0.81 | < 0.0001 |

The hazard ratios indicate that, once the adverse impact of stage on survival has been accounted for, longer duration of symptoms is a beneficial rather than an adverse prognostic factor.

date of diagnosis those with longer delays within each stage tended to have better survival, though none of the differences reached significance. When survival was measured from the onset of symptoms, these trends were more marked and reached significance among patients in stages II ($P = 0.01$), III ($P = 0.001$) and IV ($P \leq 0.001$) (Figure 3).

Because of the finding that patients with grade III tumours presented significantly earlier than others, the impact of delay within each tumour grade was assessed. For patients with ductal carcinomas, delays of 12 or more weeks in presentation had an adverse impact on survival in each tumour grade (grade I, $P = 0.05$; grade II, $P = 0.001$; grade III, $P = 0.007$).

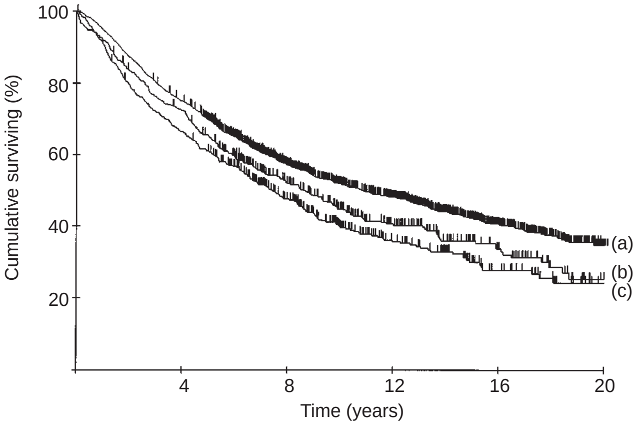

**Figure 4** Influence of 'total delay' on survival. (a) Delay < 12 weeks ($n = 1952$). (b) Delay 12–26 weeks ($n = 491$). (c) Delay > 26 weeks ($n = 494$). $P < 0.001$

### Multivariate analyses

The results of multivariate analyses of factors influencing survival from diagnosis and from onset of symptoms are shown in Tables 3 and 4. In the first analyses (Table 3), only grade, duration of symptoms, menopausal status and age were entered into the model (i.e. factors related to the extent of disease were excluded). Longer duration of symptoms had a highly significant adverse influence on survival in this model ($P < 0.0001$). When tumour size and stage were included in the model (Table 4) the adverse influence of longer duration of symptoms was no longer apparent. Indeed the hazard ratios for duration of symptoms indicate that longer duration of symptoms is a beneficial factor in these circumstances. These findings are in keeping with the effect of delay within each stage observed in the univariate analyses.

### The influence of 'total delay' on survival

The influence of total delay on survival was assessed by adding the interval between first hospital visit and first treatment for each patient to the recorded duration of symptoms at the time of first hospital visit. As shown in Figure 4 the survival pattern for groups of patients with different total delay intervals is similar to that observed for delay in presentation (Figure 1).

### DISCUSSION

This is the largest single institution study of delay and survival from breast cancer in the UK and, to the best of our knowledge, in the world. The findings of this study strongly support the primary hypothesis that longer duration of symptoms is associated with worse survival rates. Our findings are consistent with the consensus in the literature that longer delays are associated with larger tumour sizes (Fisher et al, 1977; GIVIO, 1986; Neave et al, 1990; Rossi et al, 1990) and with more advanced stage (Wilkinson et al, 1979; Elwood and Moorehead, 1980; GIVIO, 1986; Machiavelli et al, 1989; Rossi et al, 1990). In keeping with a large, multicentre study from Denmark, we found that longer duration of symptoms is associated with older age (Afzelius et al, 1994). Others have reported, however, that delay is associated with younger age (Machiavelli et al, 1989; Richardson et al, 1992).

 © Cancer Research Campaign 1999

The impact of delay on survival measured from the onset of symptoms has only rarely been assessed. Elwood and Moorehead (1980) examined cohorts of women diagnosed in Vancouver between 1945 and 1975. As with the current study, they showed that longer delays adversely affect survival not only when survival is measured from the date of diagnosis but also when survival is measured from the onset of symptoms. This finding is of considerable importance as it demonstrates that the impact of delay is not solely due to a lead-time bias (i.e. the adverse influence on survival of delays before presentation/treatment cannot simply be attributed to patients being further from the point of onset of symptoms and thus inevitably closer to the time of death).

The findings from this study indicate that patients with longer delays before their first hospital visit (either because of their own delay or because of delay in referral to hospital) tend to have tumours that are of a more advanced stage. The cancers in patients experiencing longer delays are, if anything, less biologically aggressive, as measured by the histological grade of the tumour, than those in women with shorter delays. However, the subanalyses by tumour grade show that longer delays are associated with worse survival rates irrespective of tumour grade. The multivariate analyses indicate that longer delays have an adverse impact on survival, which can be attributed to the more advanced stage of disease at presentation.

Within each stage, patients with longer delays had similar or better survival than those who presented within 12 weeks of the onset of symptoms. A plausible explanation for this is that tumours that do not progress to a more advanced stage despite delays are intrinsically less aggressive than the average for that stage. This may also help to explain some of the apparently contradictory findings in the literature. Reports published since 1970 that have shown no significant relationship between delay and survival have generally been confined to patients with early stage (operable) disease (Alderson et al, 1971; Dennis et al, 1975; Wallgren et al, 1976; Fisher et al, 1977).

The association between delays of 12 weeks or more and worse long-term survival rates measured from the onset of symptoms may have implications for malpractice litigation. The number of cases of breast cancer that result in litigation related to provider-mediated delays in diagnosis appears to be increasing markedly. In the USA, the costs of such litigation cases are now second only to those associated with injuries sustained in newborn children (Kern, 1994). Analysis of the shape of the survival curves in the current study (Figure 2) suggests that, in general, deaths that occur within the first few years of the onset of symptoms are unlikely to be related to delays in presentation or diagnosis of 12 or more weeks. Later deaths may, however, be attributable to such delays, particularly if the delay has resulted in progression of the disease to a more advanced stage.

The relationship between delay and outcome suggests that the long-term survival rates for women who present with symptomatic breast cancer could be improved if effective strategies could be developed to reduce delays in presentation and/or treatment. The data available for this study relate to the period between first detection of a symptom and first hospital visit and between first hospital visit and treatment. It was not possible to define the relative contributions of patient or general practitioner delay. If effective strategies are to be developed to reduce overall delay, it will be important to identify more closely groups of patients at high risk of delay, so that interventions (e.g. educational programmes) can be appropriately targeted. Studies from the USA have indicated that patients from ethnic minority groups (Fisher et al, 1977; Vernon et al, 1985; Richardson et al, 1992) and those who are socioeconomically disadvantaged (Richardson et al, 1992) are more likely to experience delay. The underlying reasons for delay in terms of psychological and social factors are poorly understood (Facione, 1993). Recent evidence suggests that patients whose initial symptoms do not include a lump are more likely to delay seeking medical advice and that patients who do present to their GP with symptoms other than a lump are more likely to experience a delay in onward referral (Burgess et al, 1998).

We are currently undertaking a systematic review of the literature related to delay in the diagnosis of symptomatic breast cancer. In addition to clarifying the evidence related to the impact of delay on stage and survival, the review will examine the evidence related to factors that determine patient and provider delay. The outcome of this review will inform the design of prospective studies to define who is most at risk of delay and why delays occur.

### ACKNOWLEDGEMENTS

We are grateful to Mr JL Hayward, Mr MA Chaudary and other members of the surgical team within the breast unit for ensuring that duration of symptoms was recorded at the time of first presentation. We thank Dr RR Millis for undertaking the histological assessments throughout the study period and Mr D Altman for statistical advice.

### REFERENCES

Afzelius P, Zedeler K, Sommer H, Mouridsen HT and Blichert-Toft M (1994) Patient's and doctor's delay in primary breast cancer. *Acta Oncol* **33**: 345–351

Alderson M, Hamlin I and Staunton M (1971) The relative significance of prognostic factors in breast cancer. *Br J Cancer* **25**: 646–656

Bloom HJG and Richardson (1957) Histological grading and prognosis in breast cancer. *Br J Cancer* **11**: 339–377

Burgess CC, Ramirez AJ, Richards MA and Love SB (1998) Who and what influences delayed presentation in breast cancer. *Br J Cancer* **77**: 1343–1348

Charlson ME (1985) Delay in the treatment of carcinoma of the breast. *Surg Gynecol Obstet* **160**: 393–399

Dennis CR, Gardner B and Lim B (1975) Analysis of survival and recurrence vs patient and doctor delay in treatment of breast cancer. *Cancer* **35**: 714–720

Early Breast Cancer Trialists' Collaborative Group (1992) Systemic treatment of early breast cancer by hormonal, cytotoxic or immune therapy. *Lancet* **339**: 1–15, 71–85

Elwood JM and Moorehead WP (1980) Delay in diagnosis and long-term survival in breast cancer. *BMJ* **280**: 1291–1294

Facione NC (1993) Delay versus help seeking for breast cancer symptoms: a critical review of the literature on patient and provider delay. *Soc Sci Med* **12**: 1521–1534

Feldman JG, Saunders M, Carter AC and Gardner B (1983) The effects of patient delay and symptoms other than a lump on survival in breast cancer. *Cancer* **51**: 1226–1229

Fentiman IS, Howell A, Hamed H, Lee SM, Ranson M, Wall J, Chaudary MA, Ash CM, Gregory WM, Sellwood RA and Rubens RD (1994) A controlled trial of adjuvant tamoxifen with or without prednisolene in post menopausal women with operable breast cancer. *Br J Cancer* **70**: 729–731

Fisher ER, Redmond C and Fisher B (1977) A perspective concerning the relation of duration of symptoms to treatment failure in patients with breast cancer. *Cancer* **40**: 3160–3167

GIVIO (1986) Reducing diagnostic delay in breast cancer. *Cancer* **58**(8): 1756–1761

Hainsworth P, Henderson M and Bennett R (1993) Delayed presentation in breast cancer: relationship to tumour stage and survival. *Breast* **2**: 37–41

Huguley CM, Brown RL, Greenberg RS and Clark WS (1988) Breast self-examination and survival from breast cancer. *Cancer* **62**: 1389–1396

Kern KA (1994) Medicolegal analysis of the delayed diagnosis of cancer in 338 cases in the United States. *Arch Surg* **129**: 397–404

© Cancer Research Campaign 1999 

Machiavelli M, Leone B, Romero A, Perez J, Vallejo C, Bianco A, Rodriguez R, Estevez R, Reinaldo C, Dansky C, Alvarez L, Xynos F and Rabinovich M (1989) Relation between delay and survival in 596 patients with breast cancer. *Oncology* **46**: 78–82

MacArthur C and Smith A (1981) Delay in breast cancer and the nature of presenting symptoms. *Lancet* **1**: 601–603

Neave LM, Mason BH and Kay RG (1990) Does delay in diagnosis of breast cancer affect survival? *Breast Cancer Res Treat* **15**: 103–108

Nolvadex Adjuvant Trial Organisation (1988) Controlled trial of tamoxifen as a single agent in the management of early breast cancer. *Br J Cancer* **57**: 608–611

Raabe N and Fossaa S (1996) Primary invasive breast cancer in Oslo 1980–1989: incidence and delay. *Acta Oncologica* **35**: 9–15

Rabinovich M, Vallejo C, Perez JE, Rodriguez R, Cuevas MA, Machiavelli MR, Lacava JA, Leone BA, Romero AO, Mickiewicz E, Chacon RD and Estevez RA (1993) Impact of delay to treatment upon survival in 1067 patients with breast cancer. *Int J Oncol* **2**: 197–201

Richards MA, O'Reilly SM, Howell A, George WD, Fentiman IS, Chaudary MA, Crowther D and Rubens RD (1990) Adjuvant cyclophosphamide, methotrexate and fluorouracil in patients with axillary node-positive breast cancer: an update of the Guy's/Manchester trial. *J Clin Oncol* **8**: 2032–2039

Richardson JL, Langholz F, Bernstein L, Burciaga C, Danley K and Ross RK (1992) Stage and delay in breast cancer diagnosis by race, socioeconomic status, age and year. *Br J Cancer* **65**: 922–926

Rossi S, Cinini C, Di Pietro C, Lombardi CP, Crucitti A, Bellantone R and Crucitti F (1990) Diagnostic delay in breast cancer: correlation with disease stage and prognosis. *Tumori* **76**: 559–562

Rubens RD, Armitage P, Winter PJ, Tong D and Hayward JL (1977) Prognosis in inoperable stage 3 carcinoma of the breast. *Eur J Cancer* **13**: 805–811

Rubens RD, Saxton S, Tong D, Winter PJ, Knight RK and Hayward JL (1980) Combined chemotherapy and radiotherapy for locally advanced breast cancer. *Eur J Cancer* **16**: 351–356

Rubens RD, Hayward JL, Knight RK, Bulbrook RD, Fentiman IS, Chaudary M, Howell A, Bush H, Crowther D, Sellwood RA, George WD and Howat JMT (1983) Controlled trial of adjuvant chemotherapy with melphalan for breast cancer. *Lancet* **1**: 839–843

Rubens RD, Bartelink H, Engelsman E, Haywood JL, Rotmensz N, Sylvester R, Van der Scheuren E, Papadiamantis J, Vassilaros SD, Wildiers J and Winter PJ (1989) Locally advanced breast cancer: the contribution of cytotoxic and endocrine treatment to radiotherapy. An EORTC breast cancer cooperative group trial (10792). *Eur J Cancer Clin Oncol* **25**: 667–678

Scottish Cancer Trials Breast Group and ICRF Breast Unit, Guy's Hospital, London (1993) Adjuvant ovarian ablation versus CMF chemotherapy in premenopausal women with pathological stage II breast carcinoma: the Scottish trial. *Lancet* **341**: 1293–1298

Sheridan B, Fleming J, Atkinson L, Scott G (1971) The effects of delay in treatment on survival rates in carcinoma of the breast. *Med J Aust* **1**: 262–267

van Dongen JA, Bartelink H, Fentiman IS, Lerute T, Miolet S, Oldhuis G, van der Scheuren E, Silvester R, Tong D, Winter J and van Zijl K (1991) Randomised clinical trial to assess the value of breast conserving therapy (BCT) in Stage I and Stage II breast cancer. EORTC 10801. In *Proceedings of the 5th EORTC Breast Cancer Working Conference*, Leuven, p 13.

Vernon SW, Tilley BC, Neale AV and Steinfeldt L (1985) Ethnicity, survival and delay in seeking treatment for symptoms of breast cancer. *Cancer* **55**: 1563–1571

Wallgren A, Silfersward C and Edlund G (1976) Prognostic factors in mammary carcinoma. *Acta Radiol* **5**: 1–16

Wilkinson GS, Edgerton F, Wallace HJ, Reese P, Patterson J and Priore R (1979) Delay, stage of disease and survival from breast cancer. *J Chron Dis* **32**: 365–373

 *© Cancer Research Campaign 1999*
